# Reply to Chanen (2023) ‘bringing personality disorder in from the cold: Why personality disorder is a fundamental concern for youth mental health’

**DOI:** 10.1177/10398562231175084

**Published:** 2023-06-01

**Authors:** Stephen Allison, Jeffrey CL Looi, Tarun Joseph Bastiampillai

**Affiliations:** 1065 Adelaide, SA; 2219 Canberra, ACT; Adelaide, SA

Dear Editor,

Chanen is highly critical of our paper which heightened awareness of unproven psychotherapies for adolescent borderline personality disorder (BPD).^1,2^ Our aim was to protect traumatised young people from treatments without demonstrated efficacy that might otherwise cause harm. For example, Cognitive Behavioural Therapy (CBT) is commonly used for youth BPD, but traditional CBT does not address core aspects such as self-harm and affective lability. We made three main points in our original paper:

1. The early detection of BPD is unreliable because the categorical BPD diagnosis produces heterogeneous groups with high comorbidity needing differing approaches.
2. While psychotherapies have short-term efficacy for adult BPD, these approaches have not demonstrated efficacy for adolescents.
3. Psychotherapies do not improve the long-term trajectory of BPD.^2^

Chanen^1^ does not provide convincing evidence countering these points. Nevertheless, he asserts that the Monitoring Outcomes of Borderline Personality Disorder in Youth (MOBY) trial provides proof-of-concept for the clinical staging of and early intervention for youth BPD.^3^

However, the MOBY trial is essentially encapsulated in point 2. The trial investigated the value of psychotherapy for youth BPD and found that outcomes of a median of 23 contacts of integrated, team-based speciality BPD treatment using Cognitive Analytic Therapy as a shared model were not superior to minimal intervention (a median of 3 contacts of case management and befriending, a psychotherapy control condition that consisted of 'pleasant chats' about neutral topics and enjoyable joint activities like music and sport).^3^

It is concerning that 23 contacts with a specialist youth BPD team were not superior to 3 sessions of non-specific treatment because greater amounts of specialist therapy should predict better outcomes. Given the brevity of the 3-session intervention, most of the improvements observed during the MOBY trial were probably spontaneous change and regression-toward-the-mean over the 18-month follow-up.^4^ In terms of potential harms, befriending (pleasant chats and activities) was superior to Cognitive Analytic Therapy for suicidal ideation (according to the Beck Scale for Suicide Ideation over 12 months).^3^

In conclusion, the outcomes, or rather lack of substantive evidence thereof, in the MOBY trial reinforces concerns about the value of psychotherapies for adolescent BPD. Chanen has not presented evidence or justified argumentation to discount ours.^1,2^
